# The influence of labor education participation on the subjective well-being of college students: chain mediation effect of self-efficacy and healthy lifestyle

**DOI:** 10.3389/fpsyg.2023.1255030

**Published:** 2023-11-22

**Authors:** Danwen Qiu, Jianchao Ni, Jiaqi Yang

**Affiliations:** ^1^The School of Marxism, Xiamen University, Xiamen, Fujian, China; ^2^Institute of Education and School of Aerospace Engineering, Xiamen University, Xiamen, Fujian, China; ^3^College of Chemistry and Chemical Engineering, Xiamen University, Xiamen, Fujian, China

**Keywords:** labor education participation, subjective well-being, self-efficacy, healthy lifestyle, chain mediation effect

## Abstract

**Background:**

In the process of modernization, along with economic development, intensified social competition, and increasing mental health problems such as anxiety and depression, the issue of subjective well-being has received widespread attention. The level of subjective well-being of college students also affects whether society can achieve sustainable development. In philosophy, political science, economics, sociology and other disciplines, labor is regarded as an important factor affecting subjective well-being. Labor education is an educational activity carried out by Chinese universities in recent years. This further inspires the author to think, for the college students, will the labor education received on campus have an impact on the subjective well-being? What characteristics will its impact mechanism present? What are the characteristics of the influence on subjective well-being?.

**Methods:**

This research adopts a cross-sectional design, specifically employing a random sampling approach. In this study, the questionnaire was distributed to the college’s students of 14 universities in China through the Internet. A total of 2100 questionnaires were collected.

**Results:**

This paper mainly used questionnaires to collect data, and on this basis, examined the relationship between labor education participation, self-efficacy, healthy lifestyle and subjective well-being of college students. The results showed that: (1) Labor education participation positively affected college students’ subjective well-being. (2) Self-efficacy partially mediated the relationship between labor education participation and college students’ subjective well-being. (3) Healthy lifestyle partially mediated the relationship between labor education participation and college students’ subjective well-being. (4) Self-efficacy and healthy lifestyle played a chain mediating role between labor education participation and college students’ subjective well-being.

## 1 Introduction

The pursuit of well-being is an eternal theme in the development of human society. In the process of modernization, along with economic development, intensified social competition, and increasing mental health problems, such as anxiety and depression, the issue of subjective well-being (SWB) has received widespread attention. Subjective well-being mainly refers to an individual’s overall evaluation of their quality of life based on their own set standards. It is an important psychological parameter that reflects the quality of life of individuals in a certain society and is the overall evaluation of their quality of life (Veenhoven, 1988). As a reserve force for economic and social development, the level of subjective well-being of college students also affects whether society can achieve sustainable development. Against this background, the issue of the subjective well-being of college students has received more and more attention in all walks of life. The subjective well-being of college students is an important indicator reflecting their level of mental health and quality of life (Xiaoyi and Mingwei, 2014). If the subjective well-being of college students can be maintained at a high level, this will enhance their enthusiasm for integrating into economic and social development and participating in social construction. Therefore, improving the subjective well-being of college students has become a key issue of concern in government, society, and academic circles.

In philosophy, political science, economics, sociology, and other disciplines, labor is regarded as an important factor affecting subjective well-being. Scholars believe that through labor, people can create rich material wealth and comprehensive social relationships and obtain good physical and mental health and quality abilities, which will also enhance people’s sense of well-being. Scholars often make abstract theoretical summaries on the dialectical relationship between labor and subjective well-being, without detailed discussions on the mechanisms and processes by which labor affects subjective well-being, and lack relevant empirical research. This also inspired the authors’ thinking as follows: does labor affect subjective well-being? How does labor affect subjective well-being?

Labor education is an educational activity carried out by Chinese universities in recent years. Labor education is a comprehensive conceptual category, mainly including voluntary service, social practice, job hunting and entrepreneurship, work–study program, internship training, innovation and entrepreneurship competition, cleaning and beautification of dormitory and offices, cleaning and beautification of the campus environment, and various other forms. Labor education can improve students’ comprehensive quality and physical and mental health by guiding students to work in campus study and life (Xiangbing et al., 2021). This further inspires the authors to think for college students the following: will the labor education received on campus have an impact on their subjective well-being? What characteristics will its impact mechanism present? What are the characteristics of the influence on subjective well-being? This article uses the questionnaire survey data of college students to discuss the mechanism of the impact of labor education on subjective well-being to provide some reference for academic research and university-related work.

## 2 Literature review and theoretical assumptions

### 2.1 Research on subjective well-being, labor, and labor education

Scholars in fields such as philosophy and economics have conducted extensive discussions on the issue of subjective well-being, laying a theoretical foundation for current academic research. Classical philosophers have not yet proposed the concept of “subjective well-being” but mainly start from the “good life” and focus on discussing what factors can promote human beings to approach a better life. Plato, Aristotle, Epicurus, White, and others started discussions on the aspects of virtue, virtue, senses, and satisfaction, etc., and formed results with different viewpoints. The German philosophers Marx and Engels went deep into the economic and political structure to conduct systematic research on how human beings achieve well-being. They proposed that the realization of well-being should be based on the transformation of the social system. When “all sources of collective wealth flow fully,” “each according to his ability, each according to his needs,” and “labor has become not only a means of earning a living, but also the first necessity of life itself” (Marx and Engels, 2009), individuals can achieve all-round development and gradually approach well-being. In the field of economics, scholars have argued that a good society is based on individuals trying to maximize enjoyment and self-interest, using wealth and money as measures of pleasure and pain. Bentham was the first economist to quantify bitterness and well-being, proposing that well-being is directly proportional to the stimuli and sensations an individual receives, that factors affecting people’s sensations include physiology, psychology, customs, astronomy, and geography, and that the amount of bitterness and well-being caused by these stimuli varies from person to person (Graham and Nikolova, 2015). With the rise of positivism, psychologists extended the concept of well-being to subjective well-being and joy involving all elements of life and tried to develop a diversified index system to measure well-being. Diener ED is one of the leading researchers in the field of subjective well-being. His theoretical views had a profound impact on the academic world. He proposed that subjective well-being mainly includes two basic components, namely, life satisfaction and emotional experience. Life satisfaction refers to the overall cognitive evaluation of the quality of life, that is, the degree of satisfaction with personal life in general. In contrast, emotional experience refers to the emotional experience in an individual’s life, including positive emotions such as happiness, relaxation, and satisfaction and negative emotions such as depression, anxiety, and progress. Diener also proposed that subjective well-being mainly includes three important indicators: subjectivity, integrity, and relative stability. Subjectivity means that the evaluation of well-being mainly depends on the standards set by the actors themselves. Holistic means that the evaluation of well-being is a comprehensive psychological indicator, including cognitive evaluation and emotional experience. Stability refers to the fact that a momentary happy state of mind does not necessarily lead to long-term well-being (Biswas-Diener et al., 2004). Diener’s research results have laid a theoretical and methodological basis for academic discussions on the connotation and measurement of subjective well-being.

Based on the theory and methods of research about subjective well-being, the academic community has further promoted research on the subjective well-being of college students and mainly discussed the following issues: first, the connotation, measurement tools, and indicators of subjective well-being of college students. Scholars generally agree that the subjective well-being of college students is a psychological state that reflects the overall judgment of college students on their learning and living conditions. The measurement of the subjective well-being of college students must be based on their learning and living environment, mainly involving their emotional cognition, interpersonal relationships, family satisfaction, school satisfaction, academic satisfaction, and love satisfaction (Froh, 2004; Feng, 2009; Kern et al., 2019). There are also many studies on the measurement tools of the subjective well-being of college students, mainly including the non-verbal evaluation scale of general well-being compiled by Andrews and Withey in 1976, the general well-being scale compiled by Fazio in 1977, the Well-Being Index Scale compiled by Campbell in 1976, the Affective Scale compiled by Kamman and Flett in 1983, and the International University Survey Scale compiled by Diener in 1995. This series of scales is mainly used to measure the subjective well-being of college students. The main measurement indicators include the subjective well-being of college students and personality characteristics, individual self-esteem, social support, attribution methods, value orientation, and family economic income, etc. Second, the influencing factors of the subjective well-being of college students. Scholars believe that the subjective well-being of college students is closely related to demographic characteristics, such as gender and age, but demographic variables can only explain a small part of subjective well-being, so there are few related studies. Scholars pay more attention to the influence of the social environment and psychological indicators and put forward the following views: the higher the level of social support and the better the interpersonal relationships of college students, the less dangerous behaviors they exhibit and the stronger their subjective well-being (Alves, 2022). Students with higher academic pressure have lower subjective well-being, while those with better academic performance have stronger subjective well-being (Backman-Nord et al., 2022; Gundogan, 2023). Religious belief is also considered to be an important influencing factor. College students with different religious beliefs have different perceptions of subjective well-being (Frankel and Hewitt, 1994; Saleem and Saleem, 2017). Mood, emotion, and mental health levels are closely related to the subjective well-being of college students. Students with higher emotional stability, self-esteem, self-awareness, and self-acceptance have higher subjective well-being (Xu et al., 2016; Satici, 2019). In addition, some scholars have discussed the impact of social media, such as “Facebook,” and proposed that the more social media is used, the more shy and lonely characteristics college students will have, which will reduce the subjective well-being of college students. Public emergencies, such as COVID-19, will also affect the subjective well-being of college students. Such public events increase the perceived insecurity of individuals and reduce the subjective well-being of college students (Bharti et al., 2023).

On the topic of labor education, academic circles mainly focus on the following topics: first, the value of labor education. Labor education is a distinctively practical educational activity that is different from classroom learning. It is an important way to improve students’ mental health, comprehensive quality, social interaction ability, and subjective well-being (Feiyan, 2021). Labor education helps to cultivate workers’ positive values and moral qualities, helps to improve workers’ knowledge level, technical skills, and innovation ability, and promotes the cultivation of the all-round development of college students (Xiwu, 2020). Second, the evaluation indicators of labor education. The evaluation index system of labor education mainly includes the following three aspects: the effect of students’ participation in labor education, the ability and level of teachers in labor education, and the environment and conditions for schools to carry out labor education. Among them, students’ labor literacy can be divided into the following four secondary indicators: labor values, labor emotional quality, labor knowledge and skills, and labor practice and habits. Teachers’ ability and level in labor education mainly include teachers’ labor literacy, teachers’ labor education awareness, and teachers’ labor education teaching ability; the environment and conditions for labor education in schools include the school’s philosophy and purpose, labor education-related system and operation, labor education working mechanism and resources, labor education curriculum, and labor education-related activities (Ming, 2021).

### 2.2 Research on the influence of labor education on subjective well-being

There are still limited studies on the relationship between labor education and subjective well-being. Scholars mainly propose that labor education has a positive impact on subjective well-being based on existing theoretical resources in fields such as philosophy, economics, and psychology. They mainly discuss the impact of volunteer service, as a kind of labor education activity, on subjective well-being and believe that volunteer service participation can improve subjective well-being. Handy Femida and Sealey Anthony used the survey data of the European Value Survey (Handy and Sealey, 2022) and the World Value Survey to analyze the impact of volunteer service participation on well-being. They believed that the types of service organizations and service activities volunteers participated in had an impact on subjective well-being. Borgonovi Francesca’s research confirmed that volunteering has dual functions of self-realization and risk society for participants and posited that those who participate in volunteer activities may change people’s empathy, subjective desire, and evaluation of self-status, thus improving subjective well-being (Borgonovi, 2008). Peng Liping pointed out that volunteer service participation can positively predict the subjective well-being of college students because volunteer service is an important part of altruistic behavior, which can have a positive impact on others and society. This process also enhances participants’ self-confirmation, physical and mental health, and interpersonal relationships (Xiaohui, 2018; Liping, 2022). Accordingly, this article proposes the following hypotheses:

*H1*: Participation in labor education has a positive effect on the subjective well-being of college students.

### 2.3 Research on the mediating effect of labor education on subjective well-being

#### 2.3.1 Mediating effect of self-efficacy

So, how does labor education affect subjective well-being? Through the literature review, we found that scholars pay more attention to the mediating effect of psychological indicators, such as emotion, in the relationship between volunteer activities and subjective well-being (Dayson et al., 2021). Among numerous psychological indicators, there are many research results on self-efficacy and subjective well-being. Scholars generally agree that self-efficacy can significantly improve subjective well-being. Self-efficacy is a sense of ability and self-confidence that individuals show when facing challenges in different environments, and it is a relatively stable psychological feature (Luszczynska et al., 2005). Self-efficacy has a wide range of effects on individuals’ cognition, emotion, and behavior. Self-efficacy is the result of measuring and evaluating one’s own ability, which will, in turn, regulate the individual’s choice of behavior and the amount of effort invested. It will also affect the individual’s ability to perform a specific task (Gecas, 1989). Previous studies have shown that college students with strong self-efficacy may have positive emotional states, and positive emotional states can also significantly promote subjective well-being (Schönfeld et al., 2019). Labor education activities, such as voluntary service and practical training, will also improve the quality of college students’ stress resistance, emotional stability, physical health, social skills, and empathy. Scholars have proposed that volunteering, as a labor education activity, affects subjective well-being through a chain mediation of self-efficacy and self-identity. Volunteer service, as an altruistic behavior, is beneficial for improving the psychological quality and comprehensive abilities of college students, helping to build a positive self-awareness system and thereby enhancing their subjective well-being. Therefore, labor education may affect subjective well-being through self-efficacy. Accordingly, this study believes that self-efficacy may be a potential mediating variable between labor education participation and subjective well-being and proposes the following hypothesis:

*H2*: Self-efficacy plays a mediating role between labor education participation and subjective well-being.

#### 2.3.2 Mediation effect of a healthy lifestyle

In addition, scholars have noticed that a healthy lifestyle is also a factor that affects subjective well-being. Lifestyle refers to people’s preferences in areas of daily life, such as food, clothing, housing, and transportation. In contrast, a healthy lifestyle refers to a series of behavior patterns that maintain and promote good health based on certain motivations and abilities of individuals. The measurement dimensions of a healthy lifestyle mainly include exercise, diet and nutrition, regular life, health hazards, health responsibilities, interpersonal relationships, stress management, and life appreciation behaviors (Menakaya and Menakaya, 2022). Studies have shown that unhealthy lifestyles are predictors of emotions such as anxiety and depression (Cao et al., 2021), while healthy lifestyles can improve people’s physical and mental health and improve interpersonal relationships, thus having a significant impact on subjective well-being (Maria Mateos-Lardies et al., 2022; Liqiang et al., 2023). For college students, participation in labor education means that students need to better balance the relationship between study and life. Furthermore, labor education can motivate students to get out of the classroom and exercise. During the process, students can have more friends (Yinfeng, 2017). Thus, the more they participate in labor education, the better their physical and mental quality and interpersonal relationships will be, which will also promote a healthy and regular life and improve their subjective sense of well-being (Ming, 2021). If college students lack participation in voluntary service, social practice, club activities, job hunting, and entrepreneurship, then their unplanned study and life, lack of exercise, and other unhealthy lifestyles will follow. Therefore, this study speculates that labor education will promote subjective well-being through a healthy lifestyle and puts forward the following hypothesis:

*H3*: A healthy lifestyle plays a mediating role between labor education participation and subjective well-being.

In addition, there may also be a close relationship between self-efficacy and a healthy lifestyle. A healthy lifestyle means a more reasonable arrangement and coordination of study, work, and life, which requires individuals to have a high sense of self-efficacy (Youqin, 2022). Self-efficacy not only enhances the individual’s confidence and belief in themself but also has a positive impact on the individual’s cognition, emotion, and behavior and will also promote the individual to develop a better healthy lifestyle, which in turn has a positive impact on subjective well-being. Accordingly, this study proposes the following research hypothesis:

*H4*: Participation in labor education affects the subjective well-being of college students through the chain mediation effect of self-efficacy and a healthy lifestyle.

**Figure 1:**
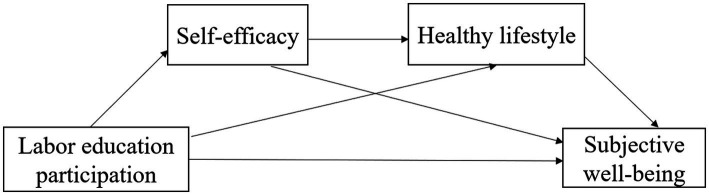
Hypothetical model.

To sum up, this study takes college students as the research object. It explores the relationship among college students’ participation in labor education, self-efficacy, healthy lifestyle, and subjective well-being. The relationship model of the four is shown in Figure 1.

## 3 Research object and method

### 3.1 Research object

This research adopts a cross-sectional design, specifically employing a random sampling approach. The questionnaire was distributed to the college students of 14 universities in China through the Internet. The inclusion of students from 14 universities enhances the generalizability of our findings to a broader student population. A total of 2,100 questionnaires were collected. To ensure the quality of the data, we established clear criteria for identifying and eliminating invalid responses. Responses were considered invalid if they exhibited the following points [specify criteria, e.g., the questionnaire takes less than 3 min; the answers provided by the respondents in the questionnaire are contradictory; respondents may provide identical answers to different questions; some questions may not have been fully answered]. The application of these criteria resulted in the exclusion of 72 questionnaires. After eliminating invalid questionnaires, 2028 valid questionnaires were obtained, with an effective rate of 98.5%. Among them, 1,019 were men, accounting for 50.2%, and 1,009 were women, accounting for 49.8%. A total of 694 were the only child in the family, accounting for 34.2%, and 1,334 were non-only children in the family, accounting for 65.8%. A total of 1,017 were registered in urban areas, accounting for 50.1%, and 1,011 were registered in rural areas, accounting for 49.8%. The average age of the respondents was 20.2 years old. It is shown that the respondents were evenly distributed in terms of demographic variables, such as gender, whether they were only children, and household registration distribution, and the sample was somewhat representative.

### 3.2 Research tools

#### 3.2.1 Labor education scale

The labor education scale was compiled by Yinfeng (2017) and Xiaodong (2021). The scale has 14 items in total, mainly measuring the participation in 14 common labor education activities, namely, volunteer service, social practice, club activities, work–study, part-time jobs, social survey, an internship in companies/units, entrepreneurial practice, labor culture festival activities, innovation and entrepreneurship competition, discipline competition, cleaning, organizing and beautifying the dormitory environment, cleaning, organizing and beautifying the office, laboratory and campus environment, labor lecture, excellent graduates’ presentation, knowledge competition, and other labor-themed education activities. The scale uses a 5-point Likert scale (0 = never, 5 = always). The higher the score of the subject on this scale, the greater their participation in labor education. The scale has a Cronbach’s alpha coefficient of 0.94.

#### 3.2.2 Self-efficacy scale

The self-efficacy scale was compiled by Wang Caikang (Caikang et al., 2001). The scale has 10 items in total. It uses a 4-point Likert scale (0 = extremely disagree, 4 = extremely agree). The higher the score of the subject on this scale, the stronger their sense of self-efficacy. The scale has a Cronbach’s alpha coefficient of 0.98.

#### 3.2.3 Healthy lifestyle scale

The healthy lifestyle scale was compiled by Youqin (2022). The scale is based on the Health Promotion Lifestyles Profile (HLP) developed by the American scholar Walker and revised by Pender. The scale has 29 items in total, including eight dimensions, namely, exercise, diet and nutrition, regular life, health hazards, health responsibility, interpersonal relationships, stress management, and life appreciation. The scale uses a 5-point Likert scale (0 = never, 5 = always), and the dimension of health hazard behavior is reverse scoring. The higher the score of the subjects on this scale, the healthier their lifestyle. The scale has a Cronbach’s alpha coefficient of 0.98.

#### 3.2.4 Subjective well-being scale

The subjective well-being scale was compiled by Margaret L. Kern (Kern et al., 2019). The scale has 20 items in total, including five dimensions, namely, engagement, persistence, optimism, connection, and happiness. The scale uses a 5-point Likert scale (0 = never, 5 = always). The higher the score of the subject on this scale, the stronger their subjective well-being. The scale has a Cronbach’s alpha coefficient of 0.99.

### 3.3 Data analysis

The SPSS24.0 statistical software was used for the common method deviation test, description, and correlation analysis. The PROCESS macro program was used for the mediation and chain mediation model test.

### 3.4 Common method bias test

Harman’s single-factor test was used for possible common method bias, and confirmatory factor analysis was performed for all items. Harman’s single-factor test found that the eigenvalues of six item factors were greater than 1, and the variance explained by the first factor was 33.6%, which was less than the critical criterion of 40%. The common variance bias in this study was not severe.

## 4 Results and analysis

### 4.1 Descriptive statistics and related analysis

Statistical analysis was carried out on the four research variables of labor education participation, self-efficacy, healthy lifestyle, and subjective well-being. The results showed that there were significant positive correlations among the variables. The significant correlation among the research variables provides a basis for the multiple mediation effect test (Table 1).

**Table 1:** The mean, standard deviation, and correlation coefficient of each variable.

	*M*	SD	1	2	3	4
1. Participation in labor education	39.62	10.39	1			
2. Self-efficacy	33.45	6.41	0.46^***^	1		
3. Healthy lifestyle	120.93	21.33	0.53^***^	0.71 ^***^	1	
4. Subjective well-being	81.63	14.98	0.47^***^	0.85^***^	0.75^***^	1

*N* = 2028, *p < 0.05, **p < 0.01, ***p < 0.001.

### 4.2 Regression and mediation effect analysis

With labor education participation as the independent variable, subjective well-being as the dependent variable, and self-efficacy and healthy lifestyle as the mediating variables, the regression analysis and mediating effect analysis were carried out. Gender and age were the two demographic variables employed as control variables. The mediation effect was tested by the bootstrap method. The sampling frequency was 5,000, the confidence interval was 99%. Model 4 from PROCESS was selected as the model.

The analysis results show that when self-efficacy is used as a mediating variable, labor education participation has a positive effect on subjective well-being (*β* = 0.14, *p* < 0.001), and self-efficacy has a positive effect on subjective well-being (*β* = 1.89, *p* < 0.001). In the mediating effect on self-efficacy, the lower limit of Bootstrap’s 99% confidence interval is 1.83, and the upper limit is 1.94, and 0 is not included in the interval, indicating that the effect has reached a significant level. Labor education participation has a positive effect on subjective well-being through self-efficacy (Table 2).

When analyzing healthy lifestyle as a mediating variable, labor education participation has a positive effect on subjective well-being (*β* = 0.15, *p* < 0.001), and healthy lifestyle has a positive effect on subjective well-being (*β* = 0.49, *p* < 0.001). In the mediating effect of healthy lifestyle, the lower limit of Bootstrap’s 99% confidence interval is 0.47 and the upper limit is 0.51, and 0 is not included in the interval, indicating that the effect has reached a significant level. Labor education participation has a positive effect on subjective well-being through a healthy lifestyle (Table 2).

**Table 2:** Regression analysis of mediation model.

	**Model 1 (dependent variable: subjective well-being)**			**Model 2 (dependent variable: subjective well-being)**	
	*β* (Boot SE)	99% Boot CI		*β* (Boot SE)	99% Boot CI
Labor education participation	0.14 (0.02)	[0.1, 0.17]	Labor education participation	0.15 (0.02)	[0.1, 0.2]
Self-efficacy	1.89 (0.03)	[1.83, 1.94]	Healthy lifestyle	0.49 (0.01)	[0.47, 0.51]
	*R*^2^ = 0.73			*R*^2^ = 0.57	
	*F* = 2750.9, *P* < 0.001			*F* = 1343.32, *p* < 0.001	

With labor education participation as the independent variable, subjective well-being as the dependent variable, and self-efficacy and healthy lifestyle as the mediating variables, the regression and mediating effects were analyzed. Gender and age were the two demographic variables employed as control variables. The mediation effect was tested by the bootstrap method. The sampling frequency was 5,000, and the confidence interval was 99%. Model 6 from PROCESS was selected as the model (Table 3).

**Table 3:** Model regression analysis.

	Equation 1 (dependent variable: self-efficacy)		Equation 2 (dependent variable: healthy lifestyle)		Equation 3 (dependent variable: subjective well-being)	
	*β* (Boot SE)	99% Boot CI	*β* (Boot SE)	99% Boot CI	*β* (Boot SE)	99% Boot CI
Gender	−2.14 (0.25)	[−2.63,-1.65]	−1.87 (0.65)	[−3.14, −0.59]	2.22 (0.33)	[1.58, 2.87]
Age	0.06 (0.02)	[0.02, 0.1]	0.15 (0.05)	[0.06, 0.24]	−0.01 (0.02)	[−0.06, 0.03]
Labor education participation	0.27 (0.01)	[0.25, 0.3]	0.52 (0.03)	[0.46, 0.59]	0.03 (0.02)	[−0.01, 0.07]
Self-efficacy			1.92 (0.06)	[1.8, 2.03]	1.52 (0.04)	[1.45, 1.59]
Healthy lifestyle					0.21 (0.01)	[0.19, 0.23]
	*R*^2^ = 0.25 *F* = 221.32, *P* < 0.001		*R*^2^ = 0.56 *F* = 632.82, *p* < 0.001		*R*^2^ = 0.77 *F* = 1379.86, *p* < 0.001	

The regression model analyzed self-efficacy, healthy lifestyle, and subjective well-being as dependent variables. The analysis results show that labor education participation has a positive effect on self-efficacy (*β* = 0.27, *p* < 0.001). Participation in labor education has a positive effect on healthy lifestyle (*β* = 0.52, *p* < 0.001). Self-efficacy has a positive effect on healthy lifestyle (*β* = 1.92, *p* < 0.001). Self-efficacy has a positive effect on subjective well-being (*β* = 1.52, *p* < 0.001) (Table 3).

It is worth noting that by analyzing self-efficacy and healthy lifestyle as mediating variables between labor education and subjective well-being separately, the two models show that labor education participation can both have a significant positive impact on subjective well-being, and self-efficacy and healthy lifestyle have a significant positive effect on subjective well-being (*p* < 0.001) (Table 3).

However, after incorporating labor education participation, self-efficacy, and healthy lifestyle into the regression model at the same time, that is, taking self-efficacy and healthy lifestyle as chain mediating valuables, the total positive effect of labor education on subjective well-being is significant (*β* = 0.66, *p* < 0.001), and the direct positive effect of labor education on subjective well-being is not significant (*β* = 0.03, *p* < 0.1). In contrast, self-efficacy has a positive effect on subjective well-being (*β* = 1.52, *p* < 0.001), and a healthy lifestyle has a positive effect on subjective well-being (*β* = 0.21, *p* < 0.001).

**Figure 2:**
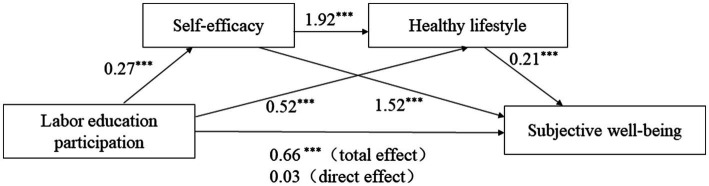
Chain mediation pattern. *p < 0.05, **p < 0.01, ***p < 0.001. The significance of the influence of labor education participation on subjective well-being is *p* < 0.1, not marked with * in the figure.

The mediation effect test shows that labor education participation affects subjective well-being through self-efficacy and healthy lifestyle. The chain mediation effect is significant. Among them, labor education participation affects subjective well-being through self-efficacy, and the mediating effect is 0.4158, accounting for 62.6% of the total effect of labor education participation on subjective well-being. Labor education participation affects subjective well-being through the mediating effect of a healthy lifestyle. The effect of size is 0.1088, accounting for 16.4% of the total effect of labor education participation on subjective well-being. Labor education participation has a chain-mediated effect on subjective well-being through self-efficacy and healthy lifestyle. The effect size is 0.1092, accounting for 16.4% of the total effect size of labor education participation on subjective well-being. The specific results are shown in Table 4 and Figure 2.

**Table 4:** Mediating effect test.

Path	Effect size	Boot SE	Boot LLCI	Boot ULCI
Participation in labor education–subjective well-being (total effect)	0.6644	0.0283	0.6089	0.7189
Participation in labor education–subjective well-being (direct effect)	0.0305	0.0182	0.0939	−0.0052
Participation in labor education–self-efficacy–subjective well-being (indirect effect 1)	0.4158	0.0283	0.3618	0.473
Participation in labor education–healthy lifestyle–subjective well-being (indirect effect 2)	0.1088	0.0126	0.0861	0.1353
Participation in labor education–self-efficacy–healthy lifestyle–subjective well-being (indirect effect 3)	0.1092	0.0127	0.0857	0.1355

Through the above data analysis, it can be found that the research hypotheses of this article are valid from h1 to h4: the more college students participate in labor education, the stronger their subjective well-being; the more college students participate in labor education, the stronger their sense of self-efficacy, which in turn improves their subjective well-being; the more college students participate in labor education, the more likely they are to choose a healthy lifestyle, which in turn improves their subjective well-being; the more college students participate in labor education, the stronger their sense of self-efficacy and the more likely it is that they choose a healthy lifestyle, which in turn improve their subjective well-being.

## 5 Conclusion and discussion

Diener E proposed that society must attach importance to subjective well-being, track, understand, and research well-being just as it does economics, and shoulder the responsibility of educating the public, scientifically understanding quality of life, and striving to create a better society (Diener et al., 1999). The pursuit of well-being has become an important issue in economic and social development and the improvement of people’s livelihood and well-being. This study focused on college students and carried out empirical research on the relationship between labor education participation and subjective well-being. It confirmed that labor education participation has a positive impact on college students’ subjective well-being, and labor education participation affects college students’ subjective well-being through self-efficacy and a healthy lifestyle.

### 5.1 The relationship between labor education participation and subjective well-being

This study found that among college students, the more they participated in labor education, the stronger their subjective well-being. Labor education is an educational and teaching activity carried out by Chinese universities in recent years. Labor education aims to lead students to participate in various practical activities, gradually establish the values of loving and respecting labor, and gradually develop good labor habits and the ideology of hard work, diligence, and thrift. In labor education activities, college students can fully integrate into various practical activities other than professional learning, which helps to improve their physical and mental health, comprehensive quality, and enhance the subject consciousness of college students. The increase in college students’ participation in labor education activities also encourages the school to create a good campus environment and atmosphere and promote the development of various undertakings in the school, which will also provide students with better learning and living conditions. The improvement of students’ physical and mental quality, labor awareness, and the optimization of the school’s overall environment are all helpful in improving the subjective well-being of college students.

### 5.2 The mediating and chain mediating effects of self-efficacy and healthy lifestyle

The impact of labor education participation on the subjective well-being of college students is mainly realized through two types of mediating variables, namely, self-efficacy and healthy lifestyle. The impact mechanism includes the following three paths.

First, labor education participation affects the subjective well-being of college students through self-efficacy, and self-efficacy plays a mediating role. It can be seen that students who are more involved in labor education are better able to assess their own abilities and qualities, regulate their own behaviors, set more reasonable goals for their studies and life, and develop more effective action strategies and coping skills for problems. All of these are conducive to college students’ improved acceptance and identification with themselves, with which their self-efficacy increases and subjective well-being also improves. These factors are conducive to the improvement of students’ self-acceptance and identity, their self-efficacy, and subjective well-being.

Second, labor education participation affects the subjective well-being of college students through healthy lifestyles, and healthy lifestyles play a mediating role. For college students, participation in labor education means that students need to better balance the relationship between study and life. The more involved in labor education, the better the students’ physical, mental, and interpersonal qualities will be, which will help improve their subjective sense of well-being.

Third, labor education participation affects subjective well-being through self-efficacy and a healthy lifestyle, and self-efficacy and healthy lifestyle play a chain mediating role in the impact of labor education on subjective well-being. The significance of the effect of labor education participation on subjective well-being decreased after including both self-efficacy and healthy lifestyle in the mediating effect model, suggesting that a large portion of the effect of labor education on subjective well-being is generated through self-efficacy and a healthy lifestyle. Participation in labor education can improve the self-efficacy of college students, and the improvement of self-efficacy will also encourage college students to arrange their studies and lives more scientifically and reasonably and enjoy a healthier lifestyle. With the improvement of self-efficacy and the development of a healthy lifestyle, college students will have more opportunities to perform well in their professional studies and extracurricular activities and will cultivate their own good overall quality, build up sufficient self-confidence, form good self-concept, and obtain harmonious interpersonal relationships, which will also contribute to the further improvement of their subjective well-being.

### 5.3 Research value

In summary, this study collected and analyzed data through a questionnaire, attempted to define labor education as a comprehensive conceptual category to examine more comprehensively college students’ participation in various practical activities other than classroom learning, and discussed the mechanism of labor education participation on college students’ subjective well-being. By confirming existing theoretical perspectives and presenting new findings, this study provides a novel perspective for universities to carry out labor education and enhance college students’ subjective well-being. College educators should focus on building a perfect working system and mechanism, carrying out more abundant labor education activities, improving the quality and attractiveness of related activities, and integrating the organizational process into the cultivation of students’ self-efficacy. In the process of organizing labor education activities, the promotion of healthy lifestyles should also be incorporated appropriately. Social work intervention techniques can be introduced, and group activities such as lectures and salons can be used to gradually improve students’ self-efficacy and promote the formation of healthy lifestyles, thus effectively enhancing college students’ subjective well-being.

### 5.4 Limitations and future directions

This study has the following limitations. First, the source of samples has certain limitations. This study was mainly carried out in Fujian Province, China, and has not yet been extended to a larger geographical space. Second, there may be some deviation in random sampling, and the accidental factors of random sampling make the structure of each unit of the sample not enough to represent the characteristics of the whole. Finally, due to the limitation of time and energy, the questionnaire of this study mainly uses the Likert scale to investigate the respondents and has not yet conducted in-depth interviews with the respondents. Future related research can try to explore more mediating variables of the impact of labor education participation on the subjective well-being of college students so as to improve the breadth of related research and provide references for universities to carry out work. Moreover, relevant research can try to use quantitative and qualitative research methods to collect various data and materials and focus on expanding the coverage of the survey to improve the pertinence and representativeness of the research. Finally, we can try to conduct comparative analysis across time periods and regions and dig out more valuable research topics through horizontal and vertical comparisons.

## Data availability statement

The raw data supporting the conclusions of this article will be made available by the authors, without undue reservation.

## Ethics statement

The present research was conducted according to the guidelines of the Declaration of Helsinki, and approved by the Ethics committee of Xiamen University. The participants provided their written informed consent to participate in this study.

## Author contributions

DQ: Conceptualization, Visualization, Writing – original draft. JN: Conceptualization, Validation, Writing – review & editing. JY: Investigation, Writing – review & editing.
